# δ-Opioid Receptor Activation and MicroRNA Expression of the Rat Cortex in Hypoxia

**DOI:** 10.1371/journal.pone.0051524

**Published:** 2012-12-13

**Authors:** Yilin Yang, Feng Zhi, Xiaozhou He, Meredith L. Moore, Xuezhi Kang, Dongman Chao, Rong Wang, Dong H. Kim, Ying Xia

**Affiliations:** 1 The Third Clinical College of Soochow University, Changzhou, Jiangsu, People’s Republic of China; 2 The University of Texas Medical School at Houston, Houston, Texas, United States of America; 3 Shanghai Research Center for Acupuncture and Meridians, Shanghai, People’s Republic of China; 4 Yale University School of Medicine, New Haven, Connecticut, United States of America; Hôpital Robert Debré, France

## Abstract

Prolonged hypoxic/ischemic stress may cause cortical injury and clinically manifest as a neurological disability. Activation of the δ-opioid receptor (DOR) may induce cortical protection against hypoxic/ischemic insults. However, the mechanisms underlying DOR protection are not clearly understood. We have recently found that DOR activation modulates the expression of microRNAs (miRNAs) in the kidney exposed to hypoxia, suggesting that DOR protection may involve a miRNA mechanism. To determine if the miRNAs expressed in the cortex mediated DOR neuroprotection, we examined 19 miRNAs that were previously identified as hypoxia- and DOR-regulated miRNAs in the kidney, in the rat cortex treated with UFP-512, a potent and specific DOR agonist under hypoxic condition. Of the 19 miRNAs tested, 17 were significantly altered by hypoxia and/or DOR activation with the direction and amplitude varying depending on hypoxic duration and times of DOR treatment. Expression of several miRNAs such as miR-29b, -101b, -298, 324-3p, -347 and 466b was significantly depressed after 24 hours of hypoxia. Similar changes were seen in normoxic condition 24 hours after DOR activation with one-time treatment of UFP-512. In contrast, some miRNAs were more tolerant to hypoxic stress and showed significant reduction only with 5-day (e.g., miR-31 and -186) or 10-day (e.g., miR-29a, let-7f and -511) exposures. In addition, these miRNAs had differential responses to DOR activation. Other miRNAs like miRs-363* and -370 responded only to the combined exposure to hypoxia and DOR treatment, with a notable reduction of >70% in the 5-day group. These data suggest that cortical miRNAs are highly yet differentially sensitive to hypoxia. DOR activation can modify, enhance or resolve the changes in miRNAs that target HIF, ion transport, axonal guidance, free radical signaling, apoptosis and many other functions.

## Introduction

Hypoxic/ischemic stress causes cortical neural injury that may result in serious neurological disorders and disability. However, scant therapeutic options are available till date. Finding new strategies to overcome this challenge has been a longstanding battle and attracted much attention of both scientists and clinicians from diverse fields. We have previously shown that the activation and/or expression of the δ-opioid receptors (DOR) is neuroprotective against hypoxic, ischemic and excitotoxic insults [1], [2], [3], [4], . In recent years, substantial evidence from various independent laboratories have reaffirmed that DOR is neuroprotective against hypoxic/ischemic stress [13], [14], [15], [16], [17], [18], [19], [20] in part by increasing the antioxidant activity [21], and partly through regulation of survival and death signaling [3], [10]. However, the exact mechanisms underlying DOR neuroprotection are still unclear.

Recent investigations suggest that microRNAs (miRNAs) play an important role in regulation of cellular survival and function. Current estimates predict that several hundred miRNAs are encoded by the mammalian genome that potentially regulates the expression of thousands of proteins [22]. There is enough evidence suggesting involvement of miRNAs in neuronal responses to hypoxic/ischemic stress. However, it is unknown if DOR neuroprotection involves regulation of miRNA expression since such an interaction between DOR and miRNAs in the brain under hypoxic conditions has not been explored previously in the published literature.

We have recently established that DOR activation alters the miRNA expression in hypoxic kidney (He et al, data to be published). Since the cortex has the highest density of DOR in the brain, we further investigated the role of miRNAs in DOR-mediated neuroprotection in the cortex. Since there is no published data on this aspect, as a first step to address this question, we examined the cortex of young adult rats exposed to prolonged hypoxia to compare miRNA changes between the cortex and kidney. Therefore, the primary purpose of this work was to determine if DOR activation affects expression of the targeted miRNAs in the cortex. Specifically, we addressed the following questions: 1) Does DOR activation affect miRNA expression profiles in the cortex under normoxia? 2) Does hypoxia at different durations differentially alter miRNA expression? 3) Does DOR activation modify hypoxia-induced changes in miRNA expression? 4) Is there a major difference between the miRNAs altered in the hypoxia-sensitive cortex and kidney?

## Results

**Figure 1:**
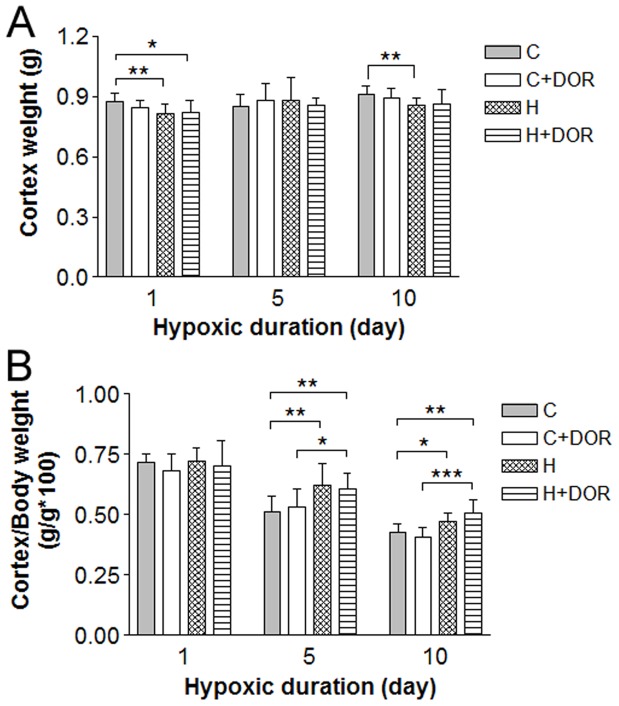
Effect of hypoxia and DOR activation on the cortex and body weight. A, Cortex weight (n = 9). B, Ratio of cortex to body weight for each study group (n = 6–9). C: control; C+DOR: control+DOR agonist UFP-512; H: hypoxia; H+DOR: hypoxia+ DOR agonist UFP-512. *p<0.05, **p<0.01, ***p<0.001. Note that small, but significant, drops in cortex weight were found in both 1-day and 10-day hypoxic animals, and the cortex/body weight ratio increased with 5–10 day hypoxia. Also note that UFP-512 did not have a detectable affect on cortex weights, but preserved more of the cortex/body weight ratio.

Exposure to a hypoxic environment consistently and significantly reduced weight gain in both UFP-512 treated and control animals beginning 24 hours after the onset of hypoxia. Small but significant drop in cortical weight was noted in both 1-day and 10-day hypoxic animals (Figure 1A). However, the cortex/body weight ratio increased with 5–10 day hypoxia, but not after 1-day exposure, suggesting that adaptive mechanisms were acting to protect the brain from atrophy during prolonged hypoxia. Although UFP-512 did not have a detectable affect on absolute cortical weights. The addition of UFP-512 preserved more of the cortex/body weight ratio compared to hypoxia exposure or UFP-512 alone (Figure 1B).

Based on microarray and PCR analysis of miRNAs altered in the hypoxic kidney (He et al, data to be published), nineteen miRNAs were selected for quantitative PCR detection in the hypoxic cortex. In addition, the effects of UFP-512 were determined over the three time points under study. As seen in the kidneys, two miRNAs, namely miR-135 and miR-199a-5p remained unaltered.

### Early Longitudinal Changes in miRNA Expression

**Figure 2:**
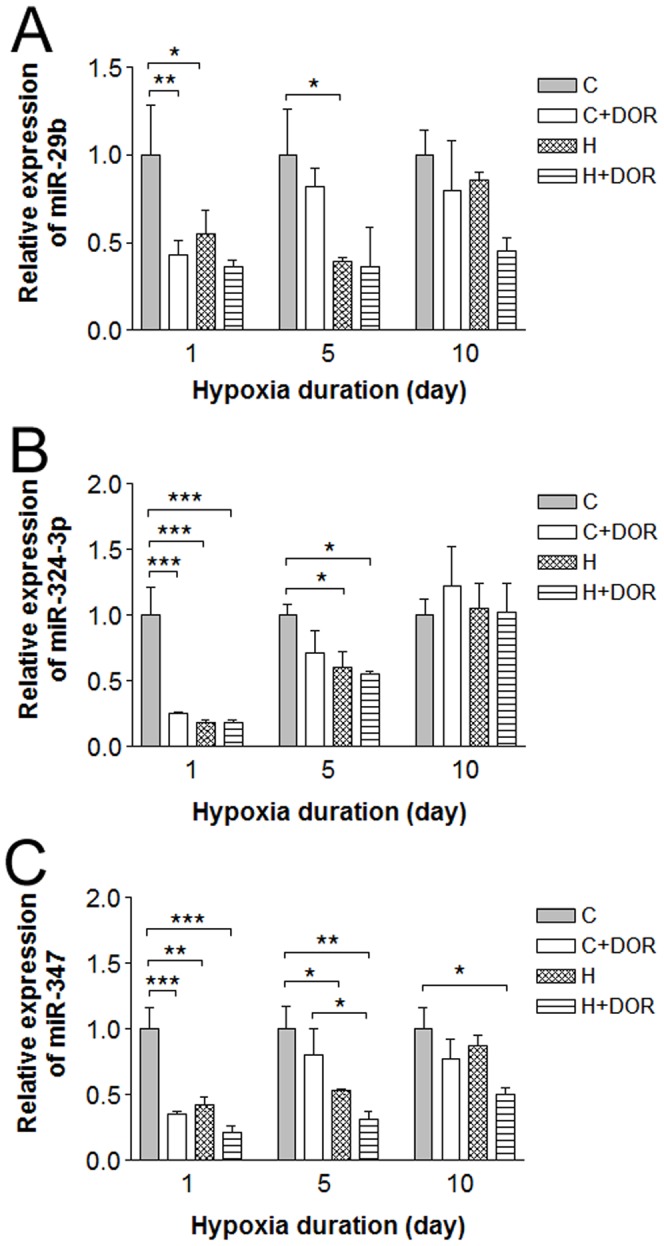
Relative miRNA expression levels of miR-29b, -324-3p and -347 in the cortex following 1, 5 or 10 days of hypoxia or UFP-512 treatment. C: control; C+DOR: control+DOR agonist UFP-512; H: hypoxia; H+DOR: hypoxia+ DOR agonist UFP-512. *p<0.05, **p<0.01, ***p<0.001. Note that miRNA expression levels of miR-29b, -324-3p and -347 were suppressed in earlier, but not later days (10-day) of hypoxia. UFP-512 suppressed these miRNAs’ expression in a similar way. These results suggest these miRNAs respond to hypoxia and DOR activation in the early period, but not in the extended duration.

Three miRNAs were significantly altered at the earliest time point. Hypoxia alone reduced the expression of miR-29b by 40% after one day and 60% after 5 days. However, miR-29b expression level almost returned to basal level after 10-day hypoxia. In the normoxic animals, a significant suppression was seen 24 hours after UFP-512 administration, while the level returned to basal level by the time of second measurement (5 days). In the hypoxic animals, UFP-512 tends to decrease the level of miR-29b, especially at the time point of 10 days, though the change was not statistically significant (Figure 2A). Similar to miR-29b, hypoxia and UFP-512 reduced miR-324-3p signals to barely 30% of the control (p<0.001). Although significant, miR-324-3p signal loss in response to 5-day hypoxia was only 40%. UFP-512 reduced the level of miR-324-3p in the normoxic animals after 5 days, but the change was not statistically significant (Figure 2B). In hypoxic animals, UFP-512 did not induce any appreciable changes at all time points (Figure 2B). In the case of miR-347, 1-day hypoxia downregulated its level by >50% and 1-day treatment with UFP-512 induced a similar reduction (Figure 2C). This reduction was also attenuated after 5 days of hypoxia or UFP-512 treatment. At Day 10, the levels of miR-347 were not significantly lower in the hypoxic animals or normoxic animals treated with UFP-512 as compared to their controls. Under hypoxic conditions, UFP-512 tends to reduce the level of miR-347 at all time points (1, 5 and 10 days). However, the differences were not statistically different yet (Figure 2C).

**Figure 3:**
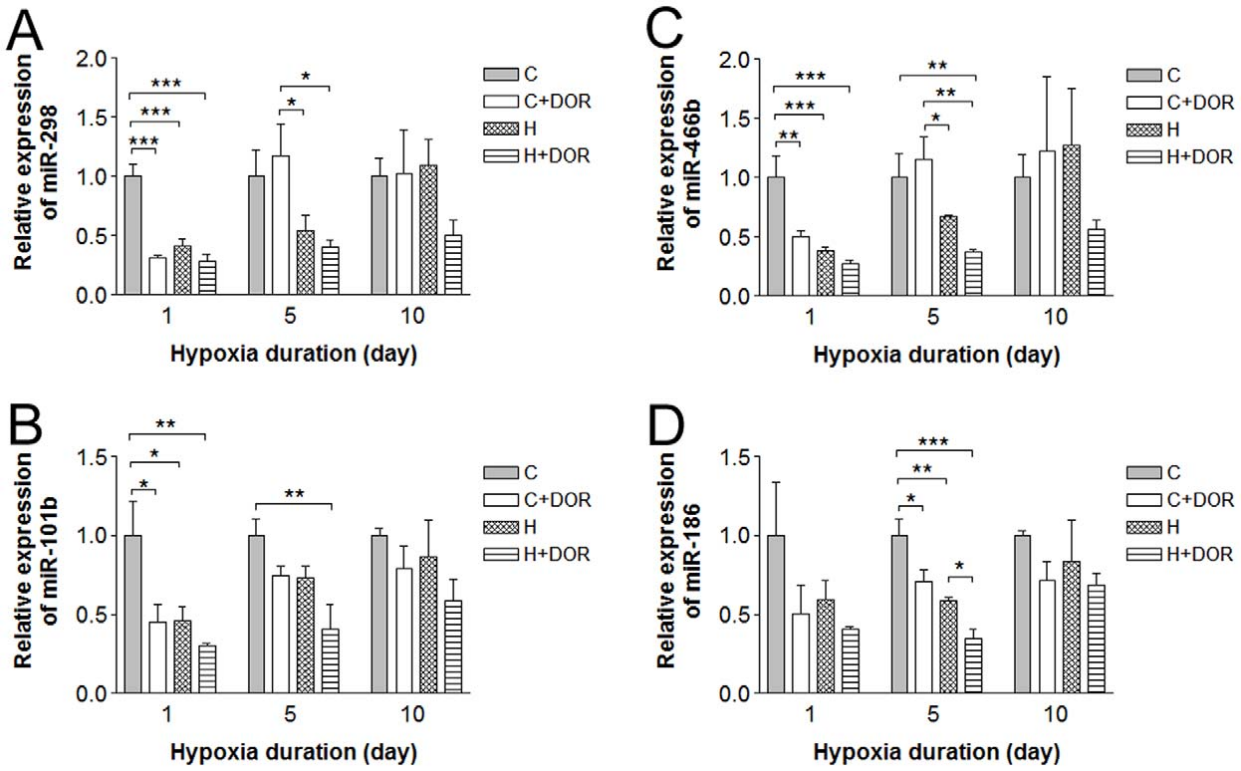
Relative miRNA expression levels of miR-298, -466b, -101b and -186 in the rat cortex following 1, 5 or 10 days of hypoxia or UFP-512 treatment. C: control; C+DOR: control+DOR agonist UFP-512; H: hypoxia; H+DOR: hypoxia+ DOR agonist UFP-512. *p<0.05, **p<0.01, ***p<0.001. Note that miRs-298, 446b and 101b are all significantly downregulated in response to hypoxia or UFP-512 alone within the first 24 hours of treatment. Hypoxia reduced the level of miR-186 after 1-day exposure and this change persisted and reached significance after 5 days of exposure to hypoxia.

Similar to the changes in miRNAs in Figure 2, miRs-298, 446b and 101b were all significantly downregulated in response to hypoxia or UFP-512 alone within the first 24 hours of treatment (Figure 3A–C). In fact, miRs-101b, -298 and -466b appeared to mimic the patterns observed with the miRNAs in Figure 2 at the first time point (1 day). However, the changes observed in the animals exposed to 5-day hypoxia are not statistically significant. Hypoxia reduced the level of miR-186 at the first time point (1 day) and this change persisted and reached significance after 5 days of exposure to hypoxia with a 40% reduction. An identical pattern was seen with UFP-512 treatment. The expression levels returned to baseline by the tenth day (Figure 3D). DOR activation with UFP-512 tends to decrease the levels of all these 4 miRNAs in the hypoxic animals at all time points (1, 5 or 10 days) though the differences were not statistically different (Figure 3D). It seems that these miRNAs respond to hypoxia and DOR activation in the early period, but not after prolonged duration.

### Late Changes in miRNA Expression

**Figure 4:**
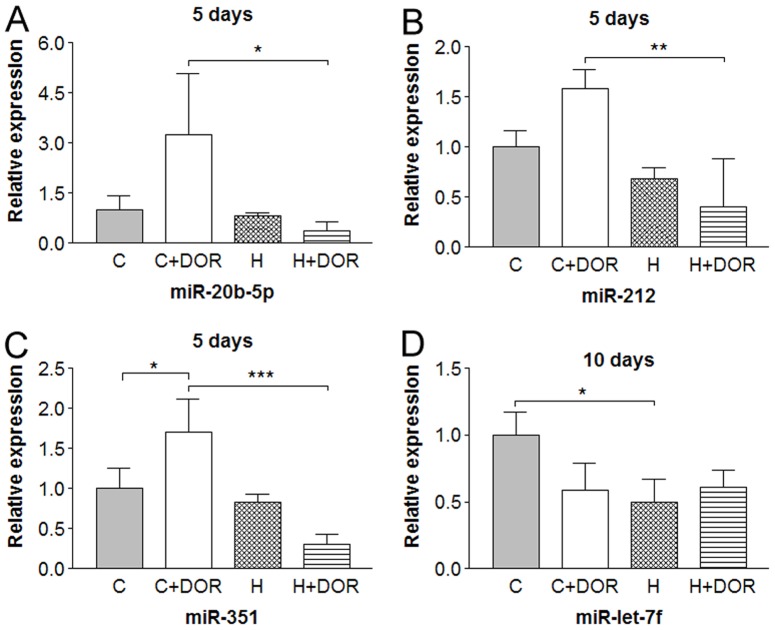
Relative miRNA expression levels of miR-20b-5p, -212, -351 and miR-let-7f in the rat cortex following 5 or 10 days of hypoxia. C: control; C+DOR: control+DOR agonist UFP-512; H: hypoxia; H+DOR: hypoxia+ DOR agonist UFP-512. *p<0.05, **p<0.01, ***p<0.001. Note that the expression of miRs-20b-5p, -212 and -351 was not significantly affected by 5 days of hypoxia alone, but significantly suppressed in the co-presence of hypoxia and UFP-512. The expression of miR-let-7f was significantly reduced by 10-day exposure to hypoxic conditions, but was not affected by UFP-512.

A third of the miRNAs demonstrated an altered expression only at a single point in time. The expression levels of miRs-20b-5p, -212 and -351 were not significantly affected by 5 days of hypoxia alone (Figure 4). In the presence of UFP-512, 5-day hypoxia produced a 6-fold reduction in miR-20b-5p as compared to the expression under normoxia in response to UFP-512 (Figure 4a). These same conditions depressed miR-212 levels by 3-fold (Figure 4b) and miR-351 by 5-fold (Figure 4c). In the normoxic cortex, UFP-512 induced a significant upregulation in miR-351 expression by over 65%, (Figure 4c), but did increase miR-20b-5p (Figure 4a) and miR-212 (Figure 4b) expressions. Chronic exposure to hypoxic conditions significantly reduced miR-let-7f by 50% after 10 days (Figure 4d). Although UFP-512 treatment showed a similar trend, this change was not statistically significant. These data suggest that these miRNAs are relatively sensitive to DOR activation, but insensitive to hypoxic stress except for miR-let-7f that was down-regulated only after a long-term (10 days) exposure to hypoxia.

### Alterations in miRNAs at Discrete Time Points

**Figure 5:**
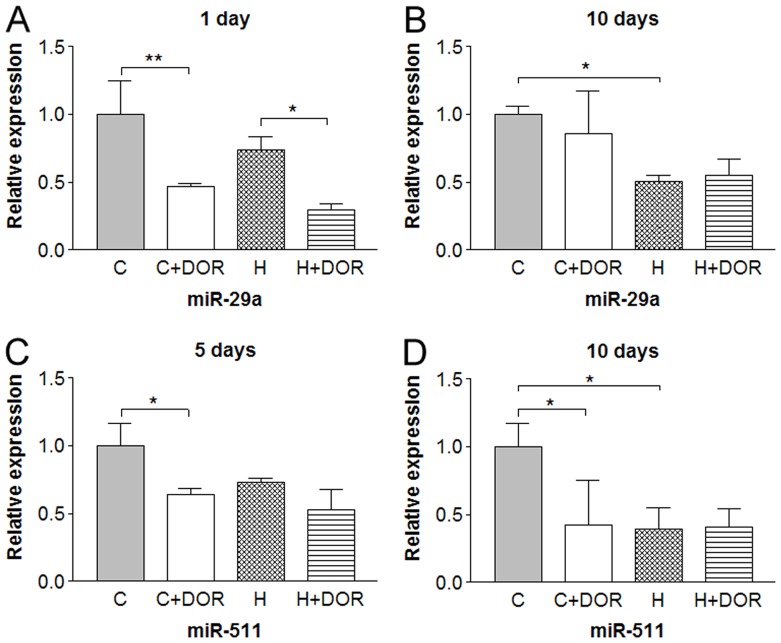
Relative miRNA expression levels of miR-29a and miR-511 in the rat cortex following 1, 5 or 10 days of hypoxia with or without UFP-512 treatment. C: control; C+DOR: control+DOR agonist UFP-512; H: hypoxia; H+DOR: hypoxia+ DOR agonist UFP-512. *p<0.05, **p<0.01. Note that miRNA expression levels of miR-29a and miR-511 were altered at discrete time points by hypoxia and/or UFP-512.

Some miRNAs were altered at discrete time points. For example, UFP-512 reduced miR-29a levels by 50% in both normoxic and hypoxic backgrounds within the first 24 hours (Figure 5a). The miR-29a expression was unaltered after 5 days (data not shown), but a significant 2-fold repression in response to hypoxia was detected after 10 days (Figure 5b). While no change in miR-511 occurred after 1 day (data not shown), UFP-512 treatment depressed miR-511 levels by 30% in the 5-day group (Figure 5c). Increasing the length of hypoxia and UFP-512 exposure to 10 days enhanced the DOR-mediated depression to 60% and revealed a hypoxia mediated 60% loss of miR-511 expression (Figure 5d).

### Alterations in miRNA Expression by the Combination of Hypoxia and DOR Activation

**Figure 6:**
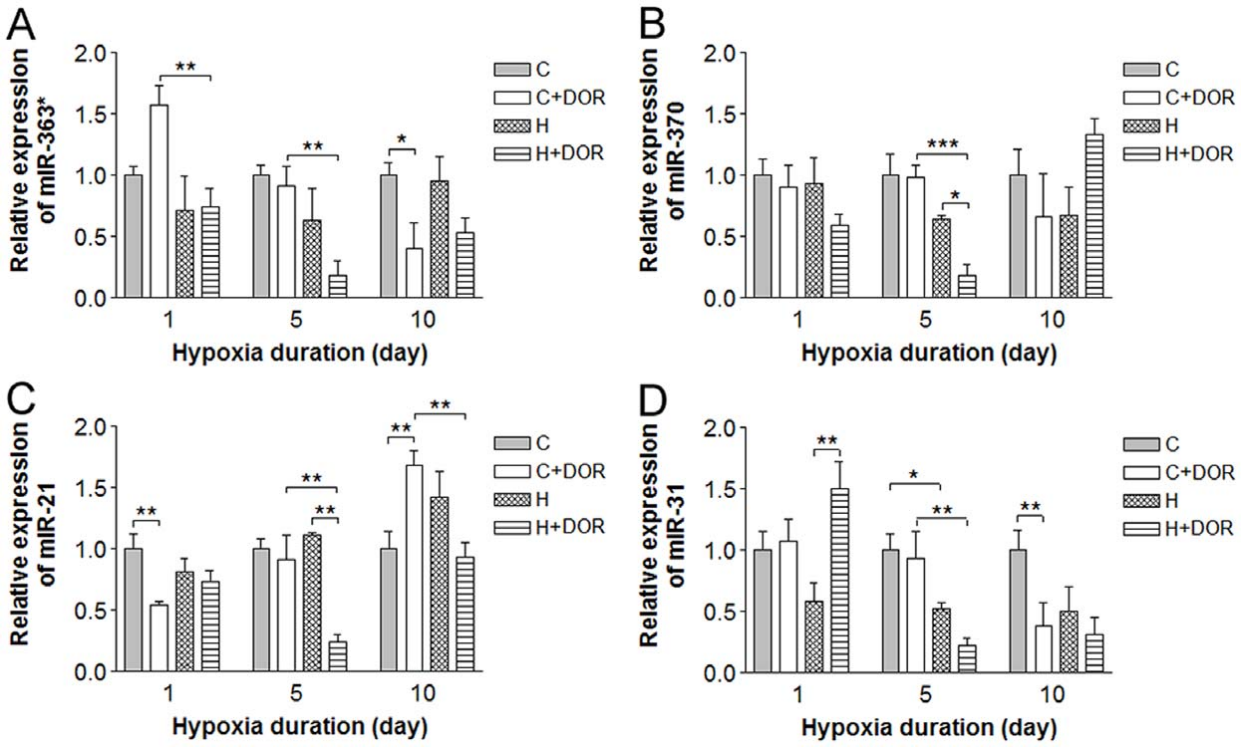
Relative miRNA expression levels of miR-363*, -370, -21 and miR-31 in the rat cortex following 1, 5 or 10 days of hypoxia with or without UFP-512 treatment. C: control; C+DOR: control+DOR agonist UFP-512; H: hypoxia; H+DOR: hypoxia+ DOR agonist UFP-512. *p<0.05, **p<0.01, ***p<0.001. Note that combination of hypoxia and UFP-512 treatment significantly altered the expression levels of miR-363*, -370, -21 and miR-31 in the cortex following 10-day treatment.

Four miRNAs exhibited significant changes when UFP-512 treatment was combined with hypoxia. The cortical miR-363* expression did not change in response to 1, 5 or 10 days of oxygen deprivation. UFP-512 exposure increased miR-363* expression within 24 hours. DOR activation significantly reduced miR-363* expression by over 70% after 5 days of hypoxia (Figure 6a). The hypoxia response in the background of DOR activation was lost after 10 days of treatment. Prolonged (10 days) UFP-512 treatment alone downregulated miR-363* expression by 50% when compared to controls, but no other statistically significant difference was noted. Although the individual treatments had no significant effect on miR-370 after 5 days, the combination downregulated its expression by 70% compared to either hypoxia or UFP-512 alone (Figure 6b). UFP-512 mediated an early 40% reduction in miR-21 within 24 hours of administration. After 5 days, this effect was lost and continued exposure resulted in a 60% upregulation of miR-21 in cortex exposed to 10-day hypoxia (Figure 6c). While hypoxia and UFP-512 individually had no effect after 5 days, the combination of two produced a dramatic 70% suppression in miR-21 expression. A similar 50% signal loss compared to UFP-512 treatment alone was observed in miR-21 after 10 days of hypoxia. Although UFP-512 treatment alone did not alter the miR-31 expression under normoxic conditions, it led to a 50% increase in miR-31 levels in the 1-day hypoxia background (Figure 6d). Following 5 days of UFP-512 treatment, miR-31 levels were unchanged in the normoxic cortex. Hypoxic conditions repressed miR-31 levels by 50 and 75% in the control and DOR activation backgrounds, respectively. Under normoxic conditions, UFP-512 had no appreciable effect on the miR-31 levels after 1 or 5 days, however, after 10 days it repressed miR-31 expression by >60%.

## Discussion

As seen in the hypoxic kidney (He et al, data to be published), oxygen deprivation in the cortex greatly alters the miRNA profiles. Owing to a higher density of DOR in the cortex, it is not surprising that the miRNAs profiled in the cortex are more affected by DOR activation with UFP-512 than in the kidney. In addition, DOR activation accelerates the resolution of hypoxia-induced changes in miR-29b, -347 and -324-3p expression in the cortex. This altered expression pattern was not observed in the kidney, suggesting a tissue specific DOR-mediated normalization. DOR-mediated and hypoxic responses were identical for miRs-466b, -101b, -298 and -186 in the cortex, but no discernible pattern was observed in the kidney. The combination of DOR activation and hypoxia dramatically downregulated miR-21 expression both in the kidney and cortex, which suggests a more generic response. These results support a potential role for miRNAs in the neuroprotective effects of DOR and suggest that elevated DOR density increases the number of miRNAs affected with differential profiles between the cortex and kidney in response to hypoxia, DOR activation or a combination of the both.

Although cultured neurons and astrocytes exposed to oxygen-glucose deprivation (OGD) exhibit diverse miRNA profiles, both cell types upregulate miR-29b in response to hypoxia [23]. However, our data demonstrates a dramatic decrease in the hypoxic brain after 1–5 day exposure, which is different from the profile of the hypoxic kidney, suggesting differences not only between cell types, between models/conditions (in vivo vs. in-vitro and hypoxia vs. OGD), but also between organs. A loss of miR-29b could increase calpain protein levels, inhibiting HIF1α and exacerbating the hypoxia-triggered flow reversal of the Na^+^/Ca^2+^ exchanger [24]. If this is the case, the recovery of miR-29b after prolonger hypoxia for 10 days could be an adaptive response; however, physiological/pathophysiological implications of miR-29b may be more complex than we can presently imagine. Its down-regulation at relatively early stage of hypoxia (e.g., after 1 or 5 days) could be a protective strategy of the hypoxic cortex in response to hypoxic stress and such adaptive ability may be impaired or lost after a prolonged hypoxia (e.g., after 10 days). Indeed, there is evidence showing that a neuroprotectant, IGF-1, downregulates miR-29b [23]. Furthermore, a recent study shows that upregulated miR-29b promotes neuronal cell death by inhibiting Bcl2L2 after ischemic brain injury [25]. The fact that DOR activation mimics the hypoxia-induced changes at day 1 provides additional support for this view. Similar changes were observed with both miRs-347 and -324-3p. Limiting the down-regulation of miR-347 and miR-324-3p can reduce the expression of calneuron-1 and mitigate the disruption of Golgi transport. However, ischemic preconditioning could down-regulate miR-324-3p [26]. Therefore, their precise role in response to hypoxia and DOR activation needs further verification.

Individually, hypoxia-induced and DOR-mediated effects on miRNAs miR-298 and miR-466b resolve at the same rate. Upregulation of miR-298 in the brain and blood has been reported in ischemia, hemorrhage and kainate seizures [27]. In contrast, the cortex in our model produces both a hypoxia- and a DOR-mediated loss of miR-298 after 1-day hypoxia. DOR activation induced a similar effect at 1-day time point. Comparable to miR-298, hypoxia and DOR activation also downregulated miR-370 and miR-363* expression after 1-day treatment. These changes in miRNA expression are predicted to release repression of expression of neuroligand and neurexophillin, ligands for neurexin, thus upregulating neurexin signaling. Neurexin is known to be upregulated after stroke and influences the N and P/Q-type Ca^2+^ channels responsible for Ca^2+^ influx during hypoxia [28]. It is also proposed that the presynaptic neurexins play a role in organizing synaptic proteins and learning related remodeling [29], [30]. Therefore, hypoxic upregulation of neurexin signals may affect ionic homeostasis and protein integrity in the cortical cells. The effect of hypoxia or DOR activation on these miRNAs is relatively short-lasting because it disappeared after a longer term of hypoxia or DOR activation (5-days or more). In sharp contrast, miR-20b-5p, -212, -351, -let-7f, and -511 did not show significant changes in their expression until 5- or 10-day exposure to hypoxia, DOR agonist, or the combination of the both. It seems that various miRNAs differentially respond to hypoxia at different stages of the stress.

It is noteworthy that DOR activation has an opposite effect on some miRNAs under normoxia vs. hypoxia. For example, DOR activation increased miR-21 under normoxic condition in the 10-day group. This DOR-mediated increase was not seen in all hypoxic groups. This suggests that DOR action may induce different effects on miRNAs depending on cellular conditions, which can have significant impact on other medical areas in addition to neuroprotection. DOR-mediated neuroprotection has been shown to be PKC-dependent [3], [5], [31]. Hyaluronan-induced PKC activation in breast cancer cells initiates signaling that culminates in the upregulation of miR-21 and the repression of the tumor suppressor, programmed cell death 4 (PDCD4) [32]. Enhancement of miR-21 expression is associated with increased cell proliferation, PDCD4 and Sprouty repression, and chemotherapy resistance in models of cardiac hypoxia preconditioning, pulmonary artery smooth muscle cells, glioblastoma and ovarian tumors [32], [33], [34], [35], [36]. Indeed, evidence shows that DOR activation increases cell proliferation [37], [38]. In the cultures, blockade of miR-21 activation with antagomir transfection reduced proliferation and increased tumor suppressor expression [32], [34], [35]. In the hypoxic models, DOR activation reduced miR-21 expression by over 70% in 5-day group. If targeted tumors express sufficient delta-opioid receptors, DOR activation could promote miR-21 down-regulation in hypoxic tumors without affecting the surrounding normal tissue. This will be an interesting area to explore in terms of potential cancer treatments.

In fact, several miRNAs, besides miR-21, showed a selective response to DOR activation under hypoxia, but not normoxia. As for instance, miR-370 had no response to DOR activation under normoxia but its expression decreased under hypoxic exposure after 5-day treatment. Castilla et al (2011) recently demonstrated that miR-370 is upregulated in the mesenchymal component of endometrial cancers undergoing epithelial-mesenchymal transition (EMT). Increased miR-370 is associated with the acquisition of a migratory phenotype and may play a role in maintaining stemness [39]. Activation of EMT is also known to increase metastatic potential in hepatocyte carcinoma [40]. DOR-mediated inhibition of miR-370 could be applied to reduce migratory capability in hypoxic tumors undergoing EMT. Interestingly, EMT can be induced by the Wnt signaling pathway, which is heavily targeted by the hypoxia and DOR sensitive miRNAs examined in this study. All but 3 of the altered miRNAs are shown to target members of the Wnt signaling pathway. Given the published roles for Wnt signaling in cell proliferation, differentiation and migration, this pathway presents ground for further investigation.

Because miRNAs regulate the expression of numerous proteins involved in various functions, the miRNAs may have a differential expression under different conditions. Also, they might respond differentially to various stresses (e.g., hypoxia vs. starvation). The alterations in their expression profiles presented in this work may limit to hypoxic condition in terms of physiological/pathophysiological impact on the body. On the other hand, we cannot distinguish the neuronal and glial profiles in the present work since the cortical tissues used here are an admixture of both cells types. We have recently found that DOR activation significantly affects astrocytic protein expression and function (Liang et al, data to be published). Therefore, DOR signing is likely to affect miRNAs in both cells. It is necessary in future study by using isolated and cultured neurons and glial cells for a better comparison and addressing this fundamental issue in understanding of DOR-mediated protection in the brain.

In summary, we have identified a subset of miRNAs that alter in response to hypoxia and/or DOR activation. These alterations in the cortical miRNA expression (with a higher density compared to kidneys) are much different from those of the kidney with more DOR-sensitive miRNAs. The changes in miRNA expression are belived to target pathways influencing ion channels/transports, HIF, axonal guidance, free radical signaling, apoptosis and many other functions. These results propose a regulatory pathway for miRNAs in neuronal responses to hypoxia and neuroprotective actions of DOR activation along with a potential role of DOR in cellular regulation that might perhaps have future implications in the cancer therapy.

## Materials and Methods

### Animals

The experiments were approved by the Review Board and the Animal Care and Use Committee of Shanghai Research Center for Acupuncture and Meridians, and were performed in accordance with its guidelines. Male Sprague Dawley rats were purchased from Shanghai Experimental Animal Center of Chinese Academy of Sciences. Three week old rats were randomly allocated to 4 study groups: control (A), DOR agonist (UFP-512) (B), hypoxia (C), and hypoxia+DOR agonist (UFP-512) (D). Groups A and B were exposed to normal air and Groups C and D were exposed to hypoxic conditions. Each group had at least 3 animals for the analysis of miRNA.

### Induction of Hypoxia

Hypoxia was induced as previously described [11], [41], [42], [43]. In brief, rats were housed in a plexiglass box (1.1 m×0.7m×0.6 m in size) with an O_2/_CO_2_ analyzer to maintain O_2_ levels at 9.5%-10% in the box. The rats in Groups C and D were placed in the hypoxic box for 1, 5 and 10 days. The box was cleaned daily in about 5 minutes while animals were removed to measure their body weights. The animals were then immediately returned to the hypoxic chamber. It took about 30 minutes after cleaning to get the oxygen level back to the desired hypoxic level.

### DOR Activation with UFP-512

The rats of Groups B and D were given intraperitoneal injection of UFP-512 (H-Dmt-Tic-NH-CH[CH2-COOH]-Bid), a potent DOR-specific agonist [6]. The injection (1 mg/kg in <1 ml) was given at day 0 (immediately before the beginning of the hypoxia) for the 24 hour group. The 5-day group received injections at day 0 and day 4, while the 10-day group received injections at day 0, day 4 and day 8. As controls, the Groups A and C received the same amount of saline at each of these time points.

### Tissue Collection

After 1, 5 or 10 days of hypoxia, the rats were anesthetized by intraperitoneal injection of 10% (400 mg/kg) chloral hydrate and decapitated. Their cortices were rapidly removed, weighed, frozen immediately in liquid nitrogen, and stored at −80°C.

### RNA Extraction and qRT-PCR

Total RNA was extracted from a 100 mg sample of frozen cortex using Trizol Reagent (Invitrogen, Carlsbad, CA, USA) according to the manufacturer’s instructions. The RNA molecules were then treated with RNase-free DNase (TaKaRa, Dalian, China) using a standardized protocol. Assays to quantify the mature miRNA were conducted as described previously [44], [45]. Briefly, 1 µg of total RNA was reverse-transcribed to cDNA using AMV reverse transcriptase (TaKaRa, Dalian, China) and looped antisense primers. The mixture was incubated at 16°C for 15 min, 42°C for 60 min and 85°C for 5 min to generate a library of miRNA cDNAs. Real-time PCR was then performed using a sequence detection system (Model 7500, Applied Biosystems, Foster City, CA, USA) with a standardized protocol. In each assay, 1 µl of cDNA (1∶50 dilution) was used for amplification. The reactions were incubated in a 96-well optical plate at 95°C for 5 min, followed by 40 cycles consisting of a 15 s anneal at 95°C and a 1-min extension at 60°C. All reactions were performed in triplicate. After the reactions were completed, the threshold cycle (Ct) values were determined using the default threshold settings and for analyzing the RT-PCR data. The ΔCt of each miRNA was determined relative to U6 RNA, an endogenous control miRNA that is robustly and invariantly expressed in all cell types. To exclude extreme outliers, miRNAs with expression lower than a preset threshold (Ct_miRNA_−Ct _U6_<15, mean fold change >2 or <0.5) were eliminated.

### Statistical Analysis

Results are presented as the mean ± SD with a minimum n = 3. The mean value for Group A (the control) for each time point is set at one. Statistical significance was determined using either a student’s t-test or a one-way ANOVA following Tukey’s Multiple Comparison Test on paired columns as appropriate.
